# Assessing the Tibial Tubercle–Posterior Intercondylar Eminence Distance as a Superior Indicator for Patellar Instability and Surgical Planning in Tibial Tubercle Osteotomy

**DOI:** 10.3390/medicina60101570

**Published:** 2024-09-25

**Authors:** Georgian-Longin Iacobescu, Antonio-Daniel Corlatescu, Octavian Munteanu, Bogdan Serban, Razvan Spiridonica, Catalin Cirstoiu

**Affiliations:** 1 Department of Orthopedics and Traumatology, University of Medicine and Pharmacy “Carol Davila”, 050474 Bucharest, Romania; georgianyak@yahoo.com (G.-L.I.); antonio.corlatescu0920@stud.umfcd.ro (A.-D.C.); bogdan.serban@umfcd.ro (B.S.); catalin.cirstoiu@umfcd.ro (C.C.); 2 University Emergency Hospital Bucharest, 050098 Bucharest, Romania; rspiridonica@yahoo.com; 3 Department of Anatomy, University of Medicine and Pharmacy “Carol Davila”, 050474 Bucharest, Romania

**Keywords:** tibial tubercle–posterior intercondylar eminence (TT-IC), diagnostic imaging, surgical guide, extensor mechanism misalignment, osteotomy

## Abstract

*Background and Objectives*: This study aimed to evaluate the tibial tubercle–posterior intercondylar eminence (TT-IC) distance as a diagnostic tool and surgical guide for correcting extensor apparatus misalignment through tibial tubercle osteotomy. *Materials and Methods*: A retrospective analysis was conducted on patients with extensor apparatus misalignment. The TT-IC distance was measured using MRI. Patients underwent tibial tubercle osteotomy, guided by the TT-IC distance for correction. Post-operative outcomes, including alignment, pain scores, and functional recovery, were assessed. *Results*: A significant correlation was found between the TT-IC distance and the degree of extensor apparatus misalignment. Utilizing the TT-IC distance as a surgical guide led to improved alignment in majority of patients. Post-operative outcomes showed reduced pain and enhanced functional recovery. *Conclusions*: The study established the TT-IC measurement as a valuable tool for determining the need for tibial tuberosity osteotomy in patients with patellar instability, particularly those with trochlear dysplasia, by providing a more precise criterion than the traditional TT-TG distance.

## 1. Introduction

The surgical indication for tibial tubercle osteotomy (TTO) is based on a tibial tubercle–trochlear groove (TT-TG) distance of 20 mm or more. This standard was proposed by Dejour et al. in 1994 [1]. In patients with trochlear dysplasia, the TT-TG distance is less consistent, especially in those with high-grade dysplasia. The TT-TG values obtained from computed tomography (CT) imaging do not always match those measured by magnetic resonance imaging (MRI), with an average difference ranging between 2.2 and 4.16 mm, according to the literature [2]. As is known, the tibial tubercle osteotomy is used in patients with patellar maltracking caused by the presence of a lateral force vector acting on the extensor apparatus. Dejour et al. highlighted in their studies that patients with a distance greater than 20 mm between the most anterior part of the tibial tubercle and the deepest part of the trochlear groove primarily require medialization of the tibial tubercle to correct the excessive lateralization of the tibial tubercle [1,3,4]. Identifying the deepest point of the trochlear groove is challenging in patients with trochlear dysplasia, especially for those with type C or D trochlear dysplasia. As highlighted by Dejour in his study, the interobserver reproducibility is only 60% [3]. Furthermore, the surgical management of patients with TT-TG values between 16 and 20 mm, who also show other signs of extensor apparatus misalignment, is challenging [1,5]. The reference value for the TT-TG distance determined by MRI is currently unknown and can be influenced by the patient’s age or body weight [6].

Patellar maltracking is closely linked to a range of histopathological alterations within the knee joint, with significant impact on the synovial tissues and fat pads. Synovitis and joint effusion are prevalent in these cases, typically presenting in the suprapatellar and infrapatellar recesses. These pathological changes are indicative of inflammatory processes and fluid accumulation, which are key contributors to pain and impaired knee function. Moreover, impingement and edema of the intra-articular fat pads, including the pre-femoral, suprapatellar, and Hoffa’s fat pads, are frequently observed. Hoffa’s fat pad, located between the patellar tendon and the tibial plateau, is particularly susceptible to inflammation—termed Hoffitis—arising from repetitive microtrauma and mechanical stress during knee motion, thereby exacerbating the clinical manifestations of patellar maltracking. Beyond the involvement of soft tissues, patellar maltracking frequently precipitates tendinopathies, especially within the quadriceps and patellar tendons. These tendinopathies are typically the result of chronic microtrauma, which leads to tendon degeneration and, in more severe instances, partial tears. This condition, commonly referred to as “Jumper’s Knee”, is prevalent among individuals who engage in repetitive jumping activities. Additionally, patellar chondropathy represents a significant histopathological consequence of maltracking. The aberrant movement of the patella leads to uneven stress distribution across the patellar cartilage, resulting in cartilage softening, fibrillation, and ultimately, chondromalacia, further intensifying knee pain and functional decline [7].

The aim of this study was to determine the correlations between a new parameter, specifically the tibial tubercle to intercondylar posterior arc distance (TT-IC), and the TT-TG distance, as well as other extensor apparatus misalignment parameters associated with trochlear dysplasia, such as the lateral trochlear slope, the trochlear angle, and trochlear facet asymmetry. The study also sought to establish a potential indication for osteotomy in these patients. This study hypothesized that the tibial tubercle–posterior intercondylar eminence (TT-IC) distance is a more reliable and reproducible parameter than the traditionally used tibial tubercle–trochlear groove (TT-TG) distance in guiding the surgical correction of extensor apparatus misalignment, especially in patients with advanced trochlear dysplasia. It is very important to establish a clearer indication for tibial tubercle osteotomy (TTO) in patients with patellar maltracking, particularly those with advanced trochlear dysplasia. Traditional criteria for TTO, based on a tibial tubercle–trochlear groove (TT-TG) distance of 20 mm or more, have limitations in patients with high-grade trochlear dysplasia, where the TT-TG measurements can be inconsistent and unreliable. Considering the variability in TT-TG values, especially in cases with complex anatomical distortions, this study explores the tibial tubercle to intercondylar posterior arc distance (TT-IC) as a potentially more reliable parameter. By examining the correlation between the TT-IC distance and other key misalignment parameters, such as the lateral trochlear slope, trochlear angle, and trochlear facet asymmetry, this study seeks to provide a more precise criterion for surgical intervention. Establishing the TT-IC distance as a potential indicator for osteotomy could offer a more effective and individualized approach to managing patellar maltracking, ultimately improving surgical outcomes in this patient population.

## 2. Materials and Methods

### 2.1. Study Participants

This is a retrospective observational study analyzing data from patients diagnosed with extensor apparatus misalignment who underwent tibial tubercle osteotomy between 2016 and 2022 at the Orthopedics and Traumatology Clinic of the Bucharest Emergency University Hospital. Within the study, data from 60 patients diagnosed with extensor apparatus misalignment, who underwent osteotomy surgery between 2016 and 2022, were analyzed. Prior approvals were obtained from the hospital’s Ethics Committee, and informed consent was secured from the patients to be included in the study cohort, ensuring adherence to international standards of research ethics and integrity. In our study, the inclusion criteria were patients diagnosed with patellar instability, recurrent luxation, and trochlear dysplasia. Patients were excluded from the study if they had prior surgeries on the knee, non-standard imaging studies, or incomplete clinical records. MRIs were taken preoperatively to accurately assess the TT-IC distance and other relevant anatomical parameters. The study included patients diagnosed with extensor apparatus misalignment and the presence of trochlear dysplasia, specifically characterized by a trochlear angle greater than 140 degrees and a lateral trochlear inclination angle below 11–12 degrees. Eligible patients had undergone tibial tubercle osteotomy between 2016 and 2022 and had available preoperative MRI and CT scans for precise measurement of the TT-PIE and TT-TG distances. Patients were required to have no prior knee surgeries and to possess complete clinical and imaging records to confirm consistency and reliability in the analysis.

All measurements were performed by the same radiologist from the Department of Radiology and Medical Imaging at the University Hospital to establish consistency and minimize inter-observer variability. The imaging assessments, including the TT-TG and TT-IC distance measurements, were conducted with each patient positioned in the supine (dorsal decubitus) position, with the knee flexed at 30 degrees. This specific positioning was chosen to provide optimal visualization of the relevant anatomical landmarks while maintaining a consistent approach across all patients. Ensuring that all measurements were taken under these standardized conditions helped enhance the accuracy and reproducibility of the radiological evaluations.

### 2.2. Surgical Procedure

The tibial tubercle osteotomy (TTO) procedure was carried out in a standardized manner to maintain uniformity across all patients. The process began with thorough preoperative planning, involving the review of detailed imaging studies, such as CT and MRI, to evaluate the tibial tubercle–trochlear groove (TT-TG) distance. This step was critical for accurately determining the extent of medialization required for the tibial tubercle.

**Figure 1:**
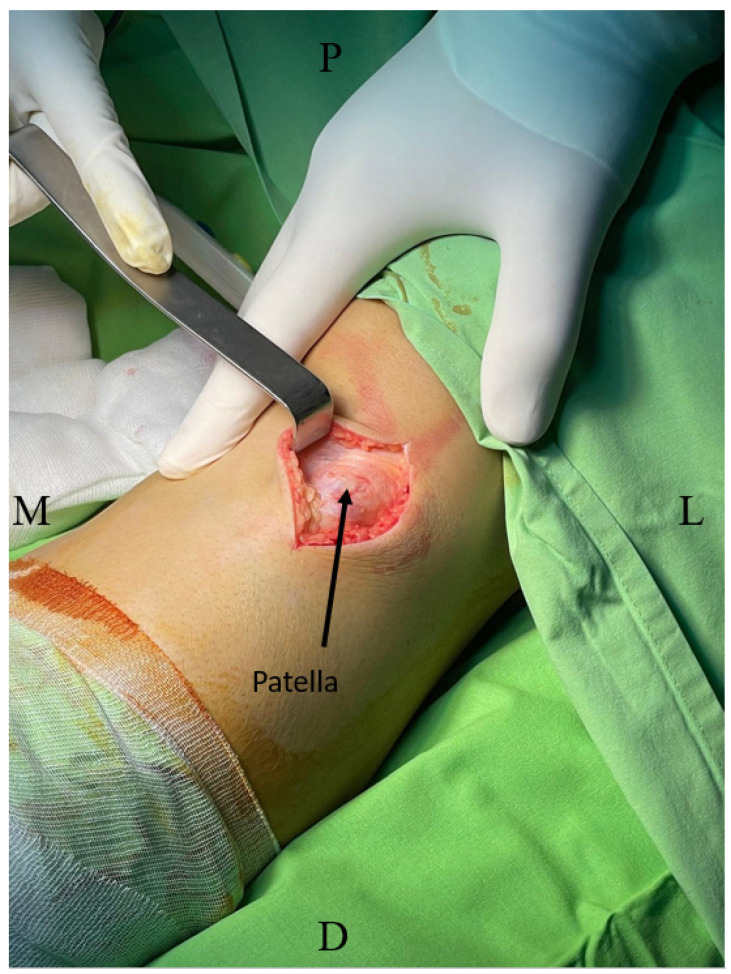
Intraoperative image showing the surgical approach (left knee). M, medial; L, lateral; D, distal; P, proximal.

Following the completion of preoperative planning, the patient was placed in a supine position on the operating table with the knee in slight flexion to allow optimal access to the surgical site. The surgical area was then aseptically prepared, and a longitudinal incision was made along the anterior aspect of the knee to expose the tibial tubercle and trochlear groove (Figure 1).

With the surgical site exposed, the osteotomy procedure commenced. The tibial tubercle was carefully cut and mobilized medially according to the preoperative plan. This repositioning was performed to correct the excessive lateralization of the tibial tubercle. After achieving the desired position, the tibial tubercle was fixed securely in place using screws to ensure stability and promote proper healing.

Following the osteotomy and fixation, the incision was meticulously closed in layers to minimize the risk of infection and promote optimal wound healing. A sterile dressing was then applied to protect the surgical site.

Post-operative care involved adhering to standard protocols, including pain management to secure patient comfort, immobilization to facilitate proper healing, and a structured rehabilitation program to restore function and strength to the operated limb. These steps collectively aimed to ensure a successful surgical outcome and a smooth recovery process for all patients.

Post-surgical rehabilitation is essential for restoring knee stability and function in patients with patellar maltracking. The initial phase focused on isometric and isotonic strengthening exercises for the quadriceps, hamstrings, and hip muscles. Isometric exercises are crucial early on, as they activate muscles without significant joint movement, thereby reducing stress on healing tissues. These exercises stabilize the knee, particularly during valgus and rotatory stresses. As recovery progresses, isotonic exercises gradually rebuild muscle strength and endurance, facilitating a transition to more dynamic activities. In the advanced rehabilitation phases, the program introduced semi-squats to enhance the strength and control of the knee extensor mechanism, with careful attention to proper alignment to avoid excessive patellar tendon stress. Progressive jump training was then incorporated to improve eccentric loading, shock absorption, and movement control, which are vital for preventing recurrent patellar dislocation. This structured approach ensures the extensor mechanism is adequately prepared for the functional demands of daily activities and sports [8].

### 2.3. Diagnostic Assessments and Measurement Techniques

**Figure 2:**
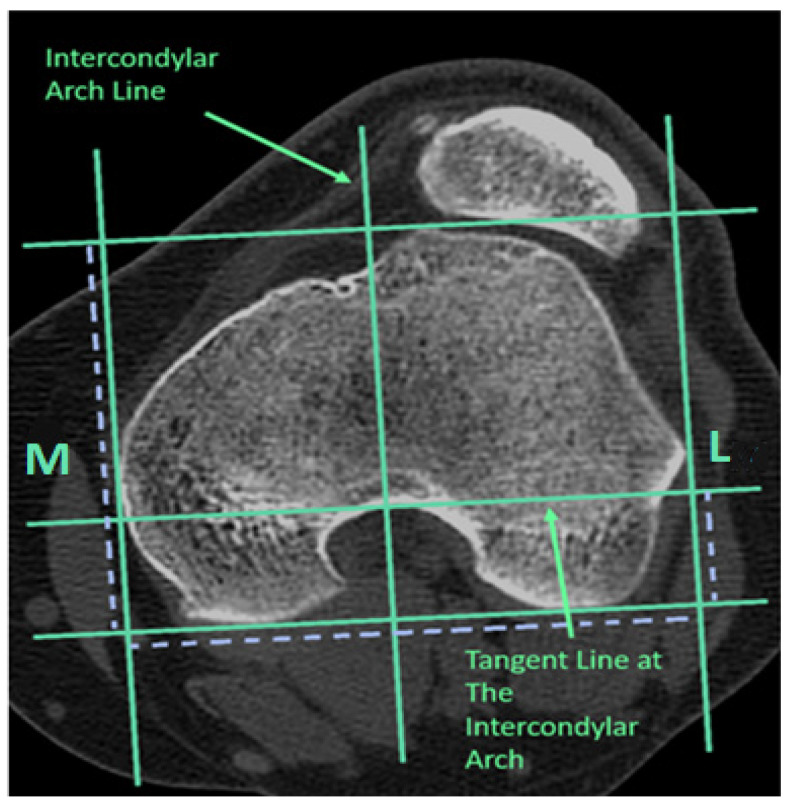
On the CT scan, the tangent line of the posterior arc, the bony landmark at the arc level, and the line of the intercondylar arc were identified (left knee). L, lateral; M, medial.

**Figure 3:**
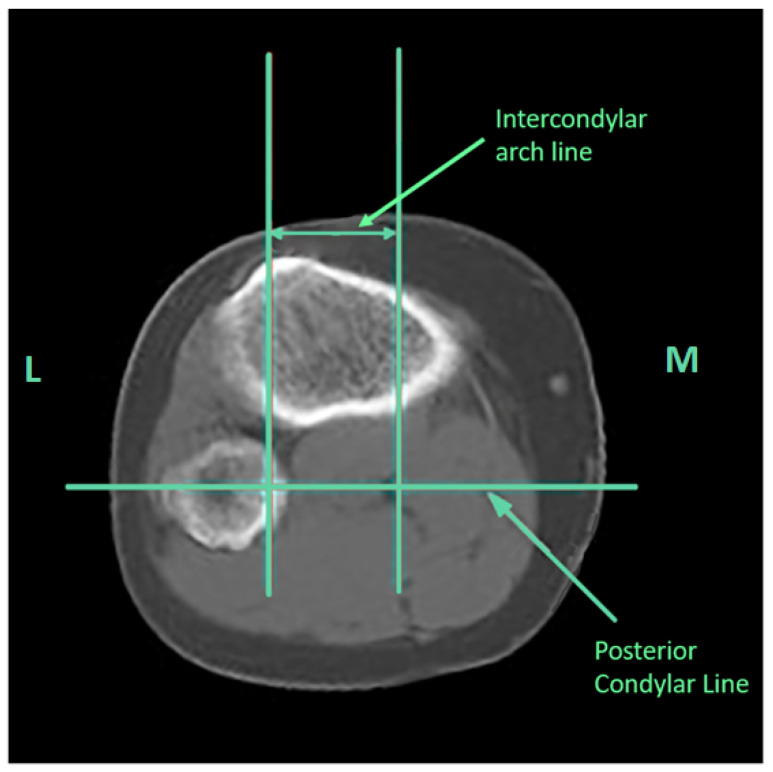
On the CT scan, the tangent line of the posterior arc, the bony landmark at the arc level, and the line of the intercondylar arc were identified (right knee). L, lateral; M, medial.

Patients were assessed using computed tomography (CT) and MRI on the operated limb. Established diagnostic parameters, such as the TT-TG distance, trochlear angle, lateral trochlear slope, and trochlear asymmetry, were determined, along with the TT-IC parameter. The measurement of the distance between the tibial tubercle and the posterior intercondylar arc was conducted as follows: A reference line was drawn tangent to the posterior femoral condyles, with a parallel line considered as the tangent line of the posterior arc, termed the bony landmark of the posterior arc. The bony landmark of the tibial tubercle was identified as the center of the tibial tubercle when the patellar tendon was in close contact with it. Consequently, the TT-IC distance was defined as the distance between two parallel lines passing through the bony edge of the tibial tubercle and the posterior arc, perpendicular to the posterior intercondylar reference line (Figure 2 and Figure 3).

All patients were assessed using high-resolution computed tomography (CT) and magnetic resonance imaging (MRI) of the operated limb. The CT scans were performed using a Siemens CT Definition Edge 128-slice scanner, with a slice thickness set to 1 mm to ensure optimal spatial resolution for detailed identification of bony landmarks. MRI assessments were conducted using a 3 Tesla Siemens Magnetom Vida scanner. For both imaging modalities, standardized patient positioning was maintained: patients were positioned supine with the knee at 30 degrees of flexion to replicate functional alignment and enhance measurement accuracy. The foot was secured in a neutral position using a foot holder to minimize movement. This imaging protocol facilitated precise and reproducible measurements of the TT-TG distance, TT-IC parameter, and other diagnostic parameters, ensuring consistent comparisons across all patients. 

### 2.4. Statistical Analysis

The statistical analysis was conducted using IBM SPSS Statistics software, version 21, utilizing a range of statistical tests tailored to the specific data characteristics and research questions. Each test employed had distinct assumptions, which were systematically verified to secure the robustness and validity of the results.

The Mann–Whitney U test was chosen to compare differences between two independent groups when the dependent variable was either ordinal or continuous but did not follow a normal distribution. This non-parametric test’s assumptions included that the two groups were independent, the dependent variable was measured on at least an ordinal scale, and the distributions of the groups were similar in shape. Independence was maintained through the study’s design, while the shape of the distributions was assessed through visual inspection of histograms to confirm comparability.

For comparing two related samples or repeated measurements within a single sample, the Wilcoxon signed-rank test was employed to evaluate whether the population mean ranks differed. The test’s assumptions required that the samples be related (paired) and that the dependent variable be measured at least on an ordinal scale. The relatedness of the samples was inherent to the study’s design, and the ordinal nature of the variable was verified during data collection to meet these assumptions.

The chi-squared test was applied to examine the association between two categorical variables. The assumptions for this test required that both variables be categorical, that observations were independent of each other, and that the expected frequency in each cell of the contingency table was at least 5. The independence of observations was ensured by the study’s design, while expected frequencies were calculated and confirmed to satisfy the necessary criteria for the test’s validity.

To measure the strength and direction of the association between two binary variables, the Phi correlation coefficient was used. This test assumes that both variables are binary and that the sample size is adequate to produce a reliable correlation. These assumptions were met by verifying the binary nature of the variables during data collection and confirming that the sample size was sufficient through a prior sample size calculation to confirm statistical power.

Across all tests, a *p*-value threshold of less than 0.05 was used to determine statistical significance. Thorough checks were conducted on all assumptions associated with each test, ensuring that the chosen statistical methods were appropriate for the data, thereby enhancing the reliability and interpretability of the study findings.

The sample size for this study was determined through a power analysis. With an effect size of 0.5, a significance level of 0.05, and a power of 0.80, we calculated that 60 patients were necessary to achieve robust statistical validity. This calculation accounted for potential dropouts and aimed to guarantee the study’s findings would be statistically significant.

## 3. Results

The analysis of our patient cohort revealed distinct demographic and clinical patterns. There was a notable predominance of female patients, with approximately 50 females compared to only 10 males, highlighting a skewed gender distribution within the study population. Age-wise, the majority of patients were young adults, predominantly in their mid-20s, with 25 years being the most represented age group (Table 1).

**Figure 4:**
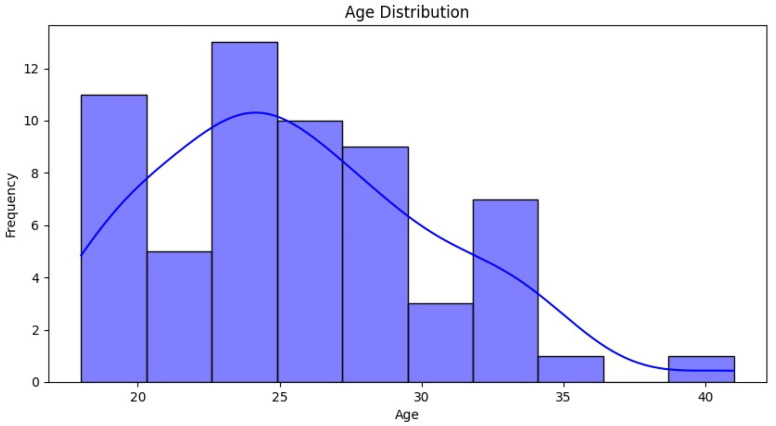
Age distribution. The ages ranged from early 20s to 40 years old. The distribution shows a higher concentration of patients in their mid-20s, with the most frequent age group being 25 years old.

**Figure 5:**
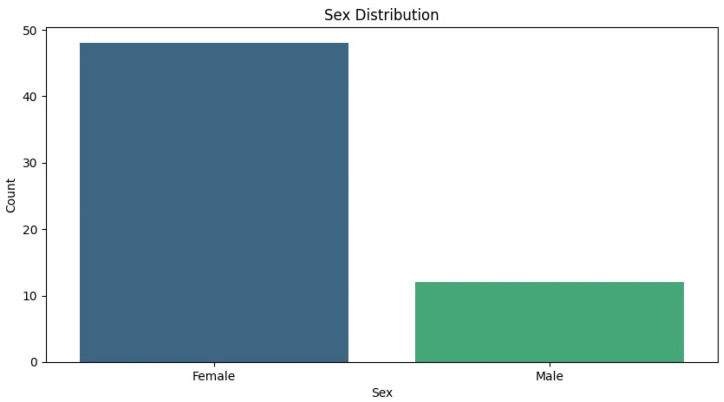
Sex distribution. A significant majority of the patients were female, with approximately 50 female patients compared to around 10 male patients.

Clinically, variations in the CT tibial tubercle–trochlear groove (TT-TG) distance were observed in relation to the trochlear angle. Patients with a trochlear angle exceeding 140 degrees demonstrated higher median TT-TG distances than those with angles of 140 degrees or less. This indicates that the trochlear angle plays a critical role in influencing the TT-TG distance, a key factor in planning tibial tubercle osteotomy. These findings underscore the importance of accounting for both demographic and anatomical factors when assessing and managing patients with patellar instability and recurrent dislocation (Figure 4 and Figure 5).

**Figure 6:**
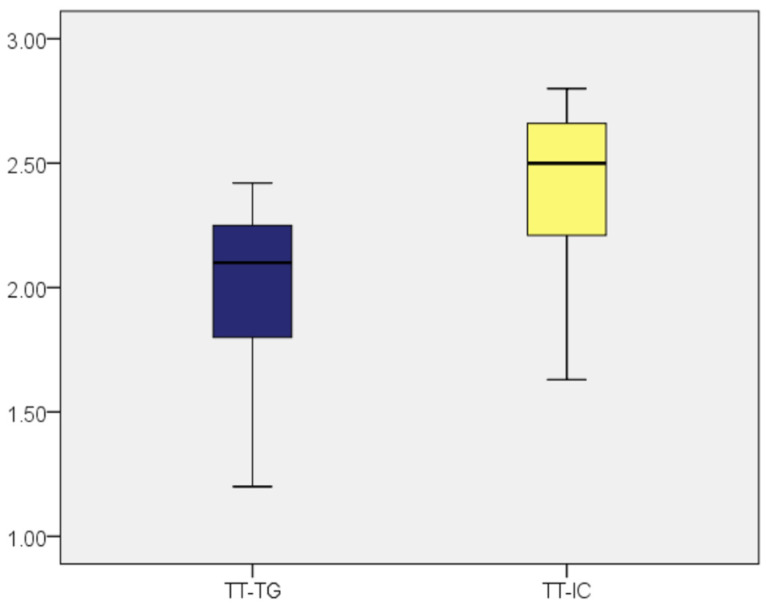
Distribution of cases based on the TT-TG and TT-IC distances measured by CT.

The average age of the patients was 25.57 years (standard deviation: 5.013; CI: [24.27; 26.86]). There were no significant differences between genders, but there was a predominantly female distribution, with 80% being women (*n* = 48) and 20% men (*n* = 12). Figure 6 shows the distribution of cases based on the TT-TG and TT-IC distances measured by CT.

The distribution of cases based on the TT-TG and TT-IC distances measured by CT showed significantly higher values for the TT-IC parameter across the entire patient group compared to the TT-TG values.

### 3.1. Assessment of the Lateral Trochlear Slope and Its Association with TT-IC by CT

The average value for the TT-IC parameter was 2.42 cm (standard deviation: 0.29; CI: [2.34; 2.49]), with a median value of 2.50 cm. Patients with a lateral trochlear slope of ≤11 degrees (measured by CT) had TT-IC values ranging from 2.10 to 2.80, with an average value of 2.4259 (standard deviation: 0.22; CI: [2.30; 2.54]) and a median value of 2.40. Meanwhile, patients with a lateral trochlear slope of >11 degrees (measured by CT) had TT-IC values between 1.63 and 2.80, with an average of 2.4202 (standard deviation: 0.31; CI: [2.32; 2.51]) and a median value of 2.54. No significant differences were observed between the TT-IC values measured in the patient group with a lateral trochlear slope of ≤11 and the group with a slope of >11 (*p* = 0.867).

**Figure 7:**
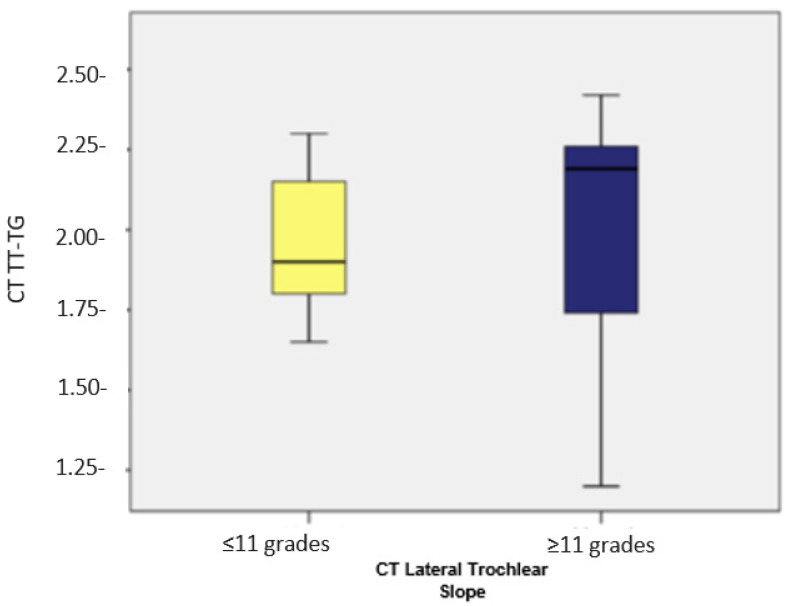
Distribution of cases based on TT-TG in patients with a lateral trochlear slope of <11 degrees and >11 degrees, respectively.

**Figure 8:**
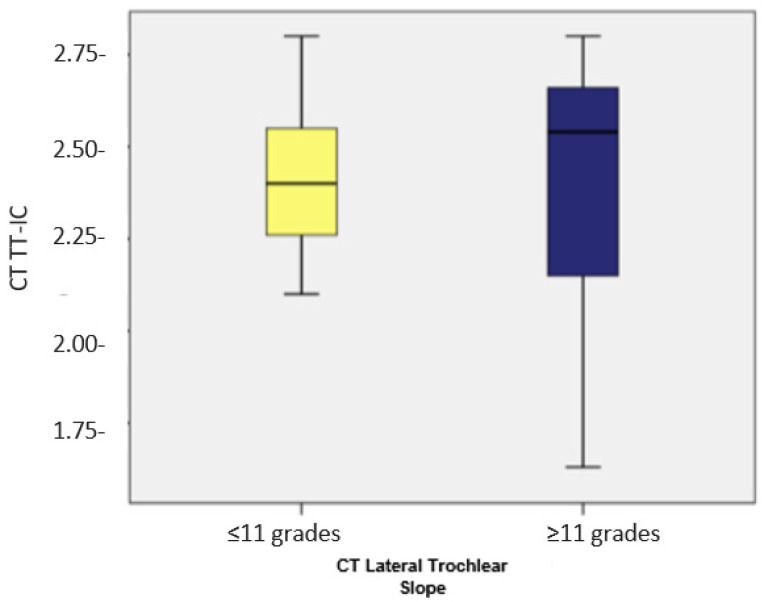
Distribution of cases based on TT-IC in patients with a lateral trochlear slope of <11 degrees and >11 degrees, respectively.

Subsequently, we compared the TT-TG and TT-IC values in patients with a lateral trochlear slope of ≤11 (*n* = 17). As previously noted, these patients showed considerably higher TT-IC distances compared to the TT-TG distances (average values: 2.42 vs. 1.95). The application of the Wilcoxon signed-rank test indicated that the differences between TT-IC and TT-TG values were statistically significant (Z = −3.626; *p* < 0.001). Significant differences between the TT-TG and TT-IC distances were also found in patients with a lateral trochlear slope of >11 (*n* = 42), with TT-IC values also being notably higher than TT-TG values (average values: 2.42 vs. 2.01; Z = −5.691; *p* < 0.001; Figure 7 and Figure 8, Table 2).

### 3.2. Assessing Trochlear Facet Asymmetry and Its Relationship with TT-TG and TT-IC by MRI

When examining trochlear facet asymmetry, patients displaying this asymmetry (as measured by MRI) had TT-TG values ranging from 1.74 to 2.30, with an average of 1.9867 (standard deviation: 0.20; CI: [1.89; 2.07]) and a median of 1.90 (*n* = 21). In contrast, patients without trochlear facet asymmetry had TT-TG values between 1.20 and 2.42, averaging 1.9886 (standard deviation: 0.37; CI: [1.84; 2.13]) with a median of 2.2 (*n* = 29). There were no statistically significant differences in TT-TG distances between patients with and without trochlear facet asymmetry (*p* = 0.510). For the TT-IC parameter measurements, patients with trochlear facet asymmetry showed values between 2.12 and 2.80, averaging 2.4248 (standard deviation: 0.21; CI: [2.32; 2.52]) with a median of 2.40. Those without this asymmetry had TT-IC values ranging from 1.63 to 2.80, with an average of 2.3972 (standard deviation: 0.36; CI: [2.25; 2.53]) and a median of 2.60. Although patients with trochlear facet asymmetry had slightly higher TT-IC values compared to those without, the differences were not statistically significant (*p* = 0.984). Subsequently, we compared the TT-TG and TT-IC values in patients with trochlear facet asymmetry (*n* = 21). As previously noted, these patients exhibited significantly higher TT-IC distances compared to the TT-TG distances (average values: 2.42 vs. 1.98). The Wilcoxon signed-rank test confirmed that the differences between TT-IC and TT-TG values were statistically significant (Z = −4.018; *p* < 0.001; Table 3).

**Figure 9:**
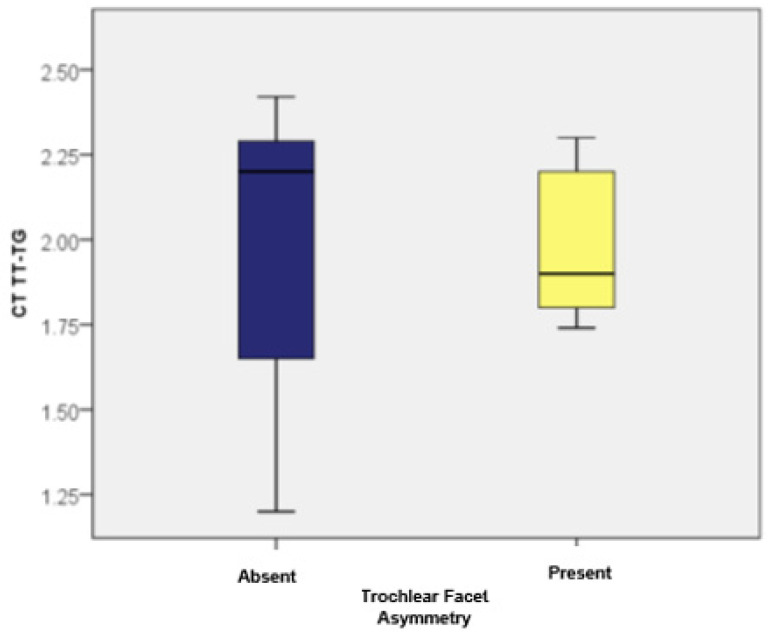
Distribution of cases based on TT-TG values in patients with and without trochlear facet asymmetry.

**Figure 10:**
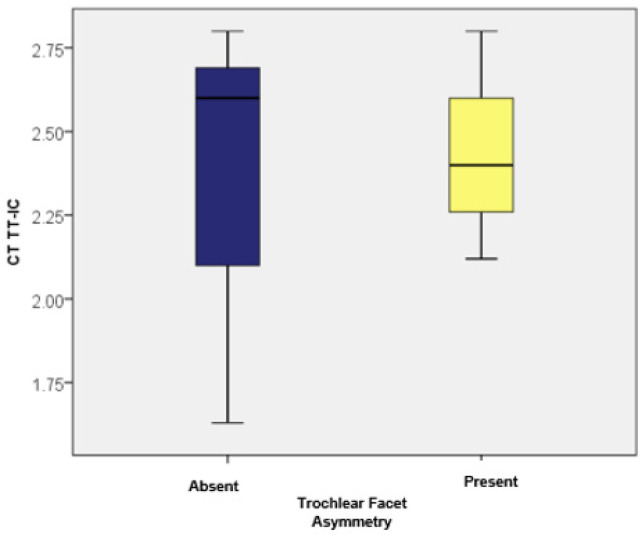
Distribution of cases based on TT-IC values in patients with and without trochlear facet asymmetry.

Similarly, significant differences between the TT-TG and TT-IC distances were observed in patients without trochlear facet asymmetry (*n* = 29). The TT-IC values were also notably higher than the TT-TG values (average values: 2.39 vs. 1.98; Z = −4.759; *p* < 0.001; Figure 9 and Figure 10).

### 3.3. Assessment of the Trochlear Angle and Its Correlation with TT-TG and TT-IC by Both CT and MRI

Patients with a trochlear angle of ≤140 degrees (measured by MRI) had TT-TG values ranging from 1.20 to 2.42, with an average of 2.02 (standard deviation: 0.36; CI: [1.87; 2.16]) and a median of 2.24 (*n* = 27). Meanwhile, those with a trochlear angle over 140 degrees had TT-TG values between 1.43 and 2.30, averaging 1.94 (standard deviation: 0.23; CI: [1.84; 2.04]) with a median of 1.90 (*n* = 23). No statistically significant differences in TT-TG distances were observed between the two groups (*p* = 0.149).

Furthermore, patients with a trochlear angle of ≤140 degrees had TT-IC values between 1.63 and 2.80, averaging 2.42 (standard deviation: 0.35; CI: [2.28; 2.56]) with a median of 2.64. In contrast, those with a trochlear angle over 140 degrees had TT-IC values ranging from 1.85 to 2.80, with an average of 2.38 (standard deviation: 0.24; CI: [2.27; 2.49]) and a median of 2.37. No significant differences in TT-IC distances were noted between the two groups (*p* = 0.425).

**Figure 11:**
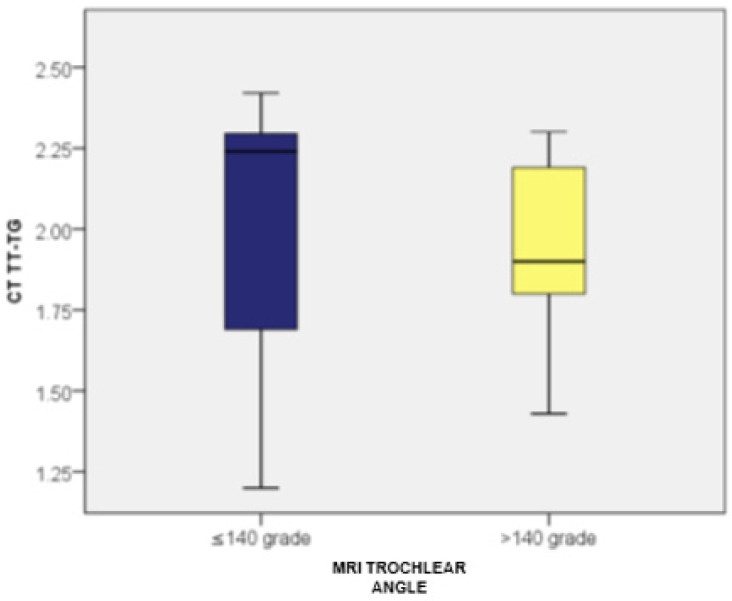
Distribution of cases based on TT-TG values and their association with the trochlear angle, determined by MRI.

**Figure 12:**
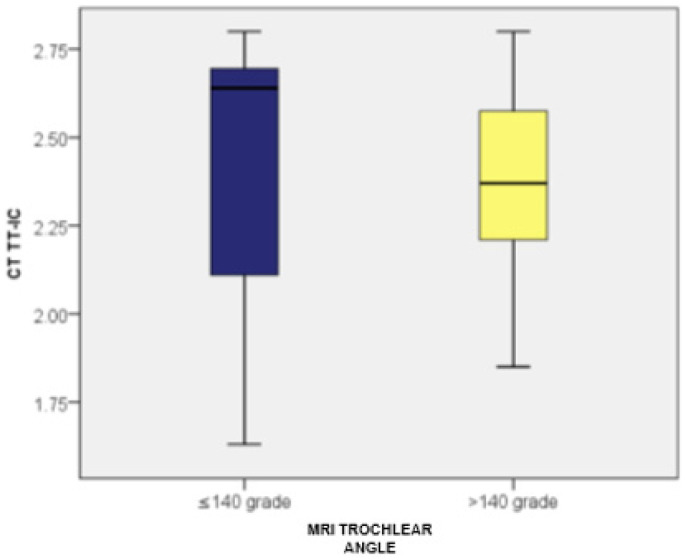
Distribution of cases based on TT-IC values and their association with the trochlear angle, determined by MRI.

By comparing the TT-TG and TT-IC values in patients with a trochlear angle of ≤140 degrees (*n* = 27), the Wilcoxon signed-rank test revealed statistically significant differences between TT-IC and TT-TG values (Z = −4.606; *p* < 0.001). This emphasized that these patients had notably higher TT-IC distances compared to TT-TG (average values: 2.42 vs. 2.02). Significant differences between TT-TG and TT-IC distances were also found in patients with a trochlear angle over 140 degrees (*n* = 23), with TT-IC values also being significantly higher than TT-TG values (average values: 2.38 vs. 1.94; Z = −4.200; *p* < 0.001; Figure 11 and Figure 12).

Regarding the CT evaluation, in the group with a trochlear angle of ≤140 degrees (measured by CT), TT-TG values ranged from 1.20 to 2.42, with an average of 2.04 (standard deviation: 0.33; CI: [1.92; 2.16]) and a median of 2.20 (*n* = 33). Meanwhile, for those with a trochlear angle over 140 degrees, TT-TG values ranged from 1.43 to 2.30, averaging 1.94 (standard deviation: 0.23; CI: [1.85; 2.03]) with a median of 1.90 (*n* = 26). No statistically significant differences in TT-TG distances were observed between the two groups (*p* = 0.056).

For the TT-IC parameter, patients with a trochlear angle of ≤140 degrees (measured by CT) had values between 1.63 and 2.80, averaging 2.45 (standard deviation: 0.32; CI: [2.33; 2.56]) with a median of 2.60. In contrast, those with a trochlear angle over 140 degrees had TT-IC values ranging from 1.85 to 2.80, averaging 2.38 (standard deviation: 0.23; CI: [2.28; 2.48]) with a median of 2.38. No significant differences in TT-IC distances were noted between the two groups (*p* = 0.184).

**Figure 13:**
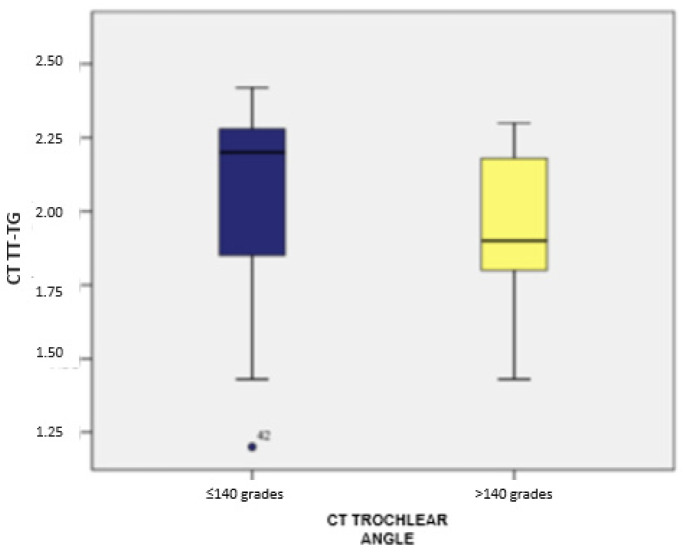
Distribution of cases based on TT-TG values and their correlation with the trochlear angle, determined using CT imaging.

**Figure 14:**
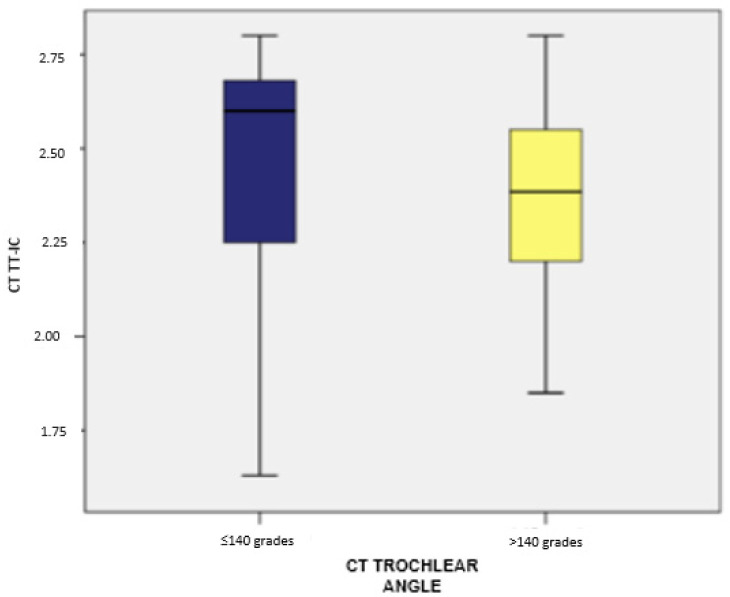
Distribution of cases based on TT-IC values and their correlation with the trochlear angle, determined using CT imaging.

Subsequently, we compared the TT-TG and TT-IC values in patients with a trochlear angle of ≤140 degrees (*n* = 33). As previously noted, these patients exhibited significantly higher TT-IC distances compared to the TT-TG distances (average values: 2.45 vs. 2.04). The Wilcoxon signed-rank test confirmed that the differences between TT-IC and TT-TG values were statistically significant (Z = −5.088; *p* < 0.001). Similarly, significant differences between the TT-TG and TT-IC distances were observed in patients with a trochlear angle over 140 degrees (*n* = 26), with TT-IC values also being notably higher than TT-TG values (average values: 2.38 vs. 1.94; Z = −4.461; *p* < 0.001; Figure 13 and Figure 14).

### 3.4. Evaluation of TT-TG and TT-IC Parameters Using ROC Curves

**Figure 15:**
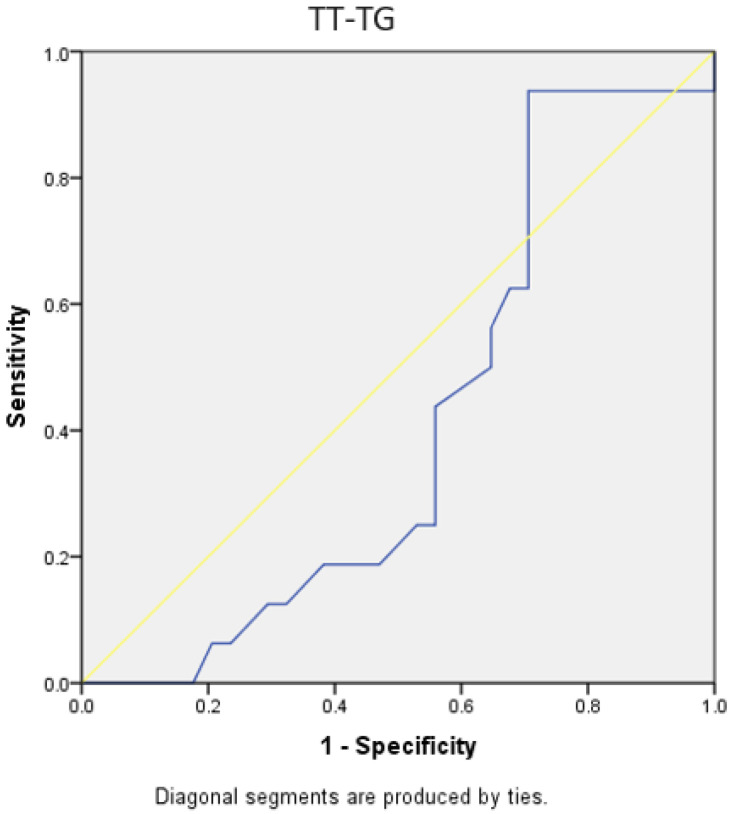
ROC curves for TT-TG distances in patients with a lateral trochlear slope of <11 degrees. The blue line represents the test’s performance at different thresholds, with a higher AUC indicating better accuracy, while the yellow diagonal shows random chance, and the goal is for the blue line to be above the yellow one, indicating the test outperforms randomness.

**Figure 16:**
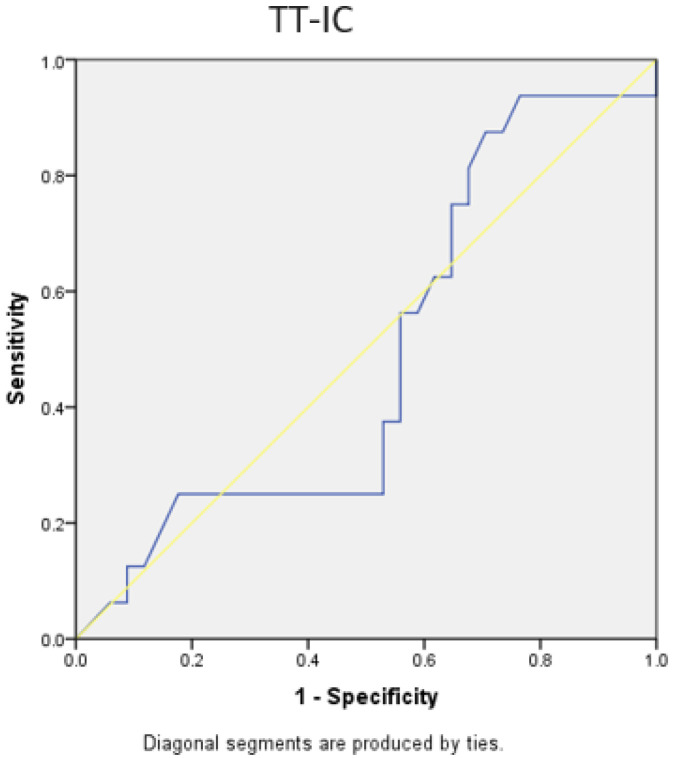
ROC curves for TT-IC distances in patients with a lateral trochlear slope of <11 degrees. The blue line represents the test’s performance at different thresholds, with a higher AUC indicating better accuracy, while the yellow diagonal shows random chance, and the goal is for the blue line to be above the yellow one, indicating the test outperforms randomness.

Both the TT-TG distance and the TT-IC distance showed limited specificity and sensitivity in predicting patients with a lateral trochlear slope of ≤11 degrees. For TT-TG, the AUC was 0.411, with a cut-off value of 1.86 cm, a sensitivity of 56%, and a specificity of 36%. For TT-IC, the AUC was 0.490, with a cut-off value of 2.25 cm, a sensitivity of 75%, and a specificity of 36%. The *p*-values were 0.313 and 0.91, respectively (Figure 15 and Figure 16).

**Figure 17:**
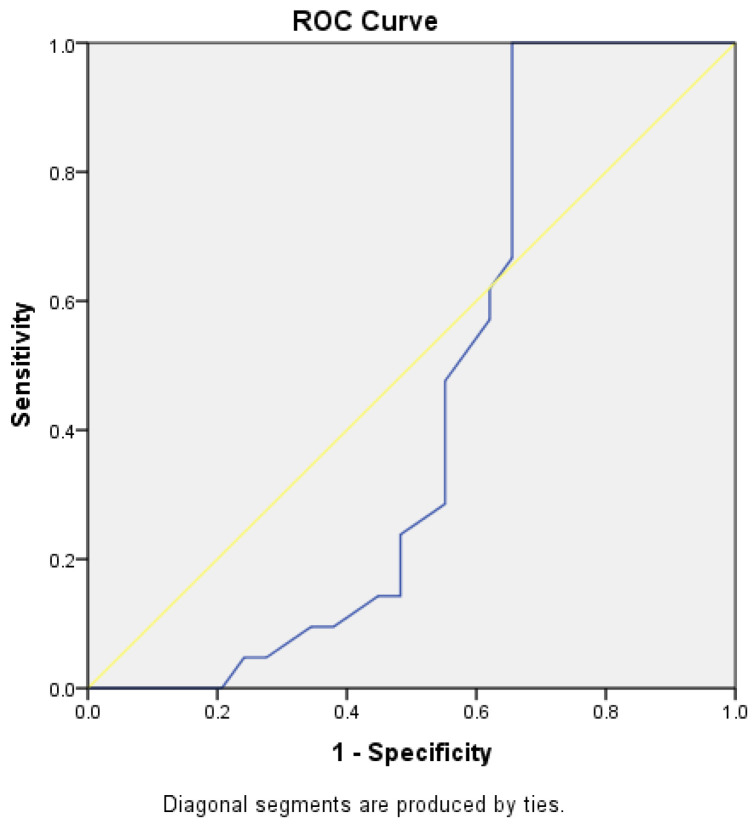
ROC curves for TT-TG distances in patients with Elmslie–Trillat osteotomy. The blue line represents the test’s performance at different thresholds, with a higher AUC indicating better accuracy, while the yellow diagonal shows random chance, and the goal is for the blue line to be above the yellow one, indicating the test outperforms randomness.

**Figure 18:**
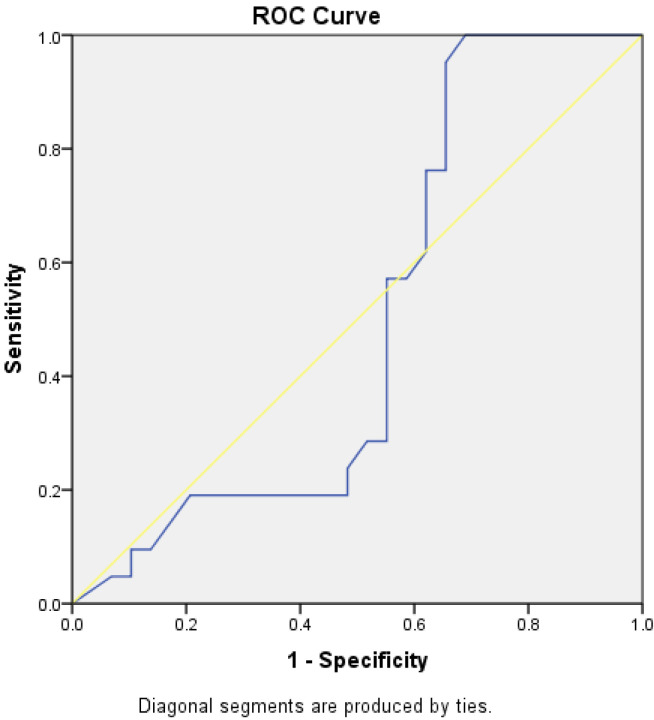
ROC curves for TT-IC distances in patients with Elmslie–Trillat osteotomy. The blue line represents the test’s performance at different thresholds, with a higher AUC indicating better accuracy, while the yellow diagonal shows random chance, and the goal is for the blue line to be above the yellow one, indicating the test outperforms randomness.

Through the analysis of ROC curves for patients undergoing Elmslie–Trillat medialization osteotomy, both the TT-TG and TT-IC parameters demonstrated high sensitivity and specificity, both statistically significant. The TT-IC distance showed slightly higher values, indicating its predictive power for this type of surgical intervention. For TT-TG, the AUC was 0.778, with a cut-off value of 1.86 cm, a sensitivity of 78%, and a specificity of 62% (*p* = 0.001). For TT-IC, the AUC was 0.787, with a cut-off value of 2.25 cm, a sensitivity of 85%, and a specificity of 62% (*p* < 0.0001; Figure 17 and Figure 18, Table 4).

## 4. Discussion

In our study, the TT-IC benchmark was compared with the traditional TT-TG distance across a cohort of patients, including those with varying degrees of trochlear dysplasia, assessed by metrics such as the lateral trochlear inclination angle, trochlear angle, and trochlear facet asymmetry, as well as those without dysplasia. The findings indicated that TT-IC values were consistently higher than TT-TG values, particularly in patients with dysplasia, suggesting that TT-IC offers greater accuracy in these cases. Both measurements were effective in predicting patellar instability; however, TT-IC demonstrated superior accuracy in the presence of dysplastic trochleae.

The clinical implications of our findings are significant for the management of patellar instability, particularly in patients with trochlear dysplasia. The TT-IC benchmark proved more accurate in assessing tibial tuberosity lateralization in advanced dysplastic cases than the conventional TT-TG distance. This is especially relevant considering the reproducibility challenges associated with TT-TG, which stem from difficulties in identifying the deepest point of the trochlear groove, particularly when the trochlear angle approaches 180 degrees, indicating a flat trochlea. Consequently, the use of TT-IC may provide more reliable data for surgical planning, such as determining the necessity for a medializing tibial tuberosity osteotomy, ultimately contributing to improved patient outcomes. 

Subsequent research has determined that an increased TT-TG distance is a predictive marker for patellar instabilities, with a value exceeding 20 mm being the gold standard for formally indicating a medializing tibial tuberosity osteotomy [9]. However, this axial measurement has shown limitations due to its reduced reproducibility, especially in patients with trochlear dysplasia, particularly those with advanced trochlear dysplasia with a trochlear angle nearing 180 degrees—a flat trochlea. This challenge arises from the difficulty in accurately determining the deepest point of the trochlear groove [10,11,12]. Consequently, there was a need for a new benchmark that could be more effective in determining the lateralization of the tibial tuberosity in patients with advanced trochlear dysplasia.

While our study introduced the TT-IC benchmark as a novel approach, it is essential to recognize that the use of axial measurements for surgical guidance is not a new concept. Previous studies have established that an increased TT-TG (tibial tubercle–trochlear groove) distance serves as a predictive marker for patellar instability and a primary indicator for medializing tibial tuberosity osteotomy. Despite its status as the gold standard, TT-TG has notable limitations, particularly in patients with advanced trochlear dysplasia, prompting the need for alternative benchmarks. Our research addressed these limitations by proposing the TT-IC benchmark, which aims to enhance measurement reliability in specific patient groups.

According to a study by Xu et al., the TT-RA (tibial tubercle–Roman arch) distance was proposed as a more reliable measure for assessing tibial tubercle positioning in patients with patellar dislocation, particularly in cases of trochlear dysplasia. Their findings demonstrated that the TT-RA distance had higher reproducibility than the traditional TT-TG (tibial tubercle–trochlear groove) distance, which is often unreliable due to the difficulty in accurately identifying the deepest point of the trochlear groove in dysplastic trochlea. Xu et al. established a surgical threshold for the TT-RA distance at 26 mm, and they reported excellent intraclass correlation coefficients (ICCs) across all Dejour classifications, indicating its utility for clinical decision-making. Furthermore, the TT-RA distance showed superior diagnostic accuracy over the TT-TG distance, especially in complex trochlear morphologies, supporting its use as a preferred surgical guide [13].

Our findings align with Xu et al.’s conclusion regarding the limitations of the TT-TG distance in patients with advanced trochlear dysplasia and the need for more reliable measurements. However, while Xu et al. recommend the TT-RA distance, our study introduced the TT-IC benchmark, which also demonstrated greater accuracy and reliability, particularly in severe dysplastic cases. Both studies emphasized the necessity of consistent and reproducible metrics across varying trochlear types, but our research further suggested that the TT-IC may offer additional advantages in evaluating tibial tuberosity lateralization in complex presentations [13]. Compared to other axial measurements, the TT-IC distance provides a clearer indication for surgical intervention in patients with complex trochlear dysplasia, as it is less affected by the variability in the trochlear groove’s anatomy. This enhances its reproducibility and reliability, particularly in severe dysplastic cases, where the traditional TT-TG distance is less dependable. Our study offered an innovative perspective by introducing a benchmark that considers anatomical variations more comprehensively, potentially improving patient outcomes through better-informed surgical decisions.

The study had its limitations. The first was that the sample size was relatively small, especially when considering patients with high-grade trochlear dysplasia, where the new benchmark showed enhanced accuracy. Second, the study was conducted at a single center, which may limit the applicability of the findings to other populations or clinical settings with different patient demographics or surgical practices.

Future research should address the limitations we found in our study. One key area is to increase the sample size, especially by including more patients with high-grade trochlear dysplasia, to better confirm the accuracy of the TT-IC benchmark. Additionally, it would be beneficial to include anatomical changes at the tibia level in the analysis to provide a more comprehensive understanding of extensor apparatus misalignments. Long-term studies could also help assess the clinical outcomes of using TT-IC for surgical planning and management of patellar instability. By addressing these aspects, future research can improve the diagnostic and treatment methods for patients with patellar instability and trochlear dysplasia.

## 5. Conclusions

This study laid the groundwork for a new axial measurement that quantifies the degree of tibial tuberosity lateralization in patients with patellar instability. Upon analysis, the new TT-IC benchmark showed higher values compared to TT-TG across all patients, with notably higher values in those with trochlear dysplasia. Therefore, when assessing a patient with patellar instability and trochlear dysplasia, it is essential to consider the TT-IC value alongside the traditional TT-TG distance to accurately determine the need for tibial tuberosity osteotomy. To address the potential indication for osteotomy, our study suggested that the new TT-IC measurement can serve as a critical factor in decision-making for tibial tuberosity osteotomy in patients with patellar instability, particularly in those with concomitant trochlear dysplasia. By demonstrating that the TT-IC values were consistently higher than TT-TG values, especially in patients with trochlear dysplasia, our findings highlighted the importance of incorporating TT-IC into the assessment process. This addition can help identify candidates who may benefit most from osteotomy, providing a clearer, more precise criterion for surgical intervention and improving patient outcomes.

## Figures and Tables

**Table 1:** Demographic characteristics of the study population.

Characteristic	Number (*n*)	Percentage (%)
Total Patients	60	100
Sex Distribution		
Female	50	83.3
Male	10	16.7
Age Distribution		
20–24 years	15	25
25–29 years	30	50
30–34 years	10	16.7
35–40 years	5	8.3
Mean Age (years)	25.57	
Standard Deviation	5.01	

**Table 2:** CT and MRI measurements by trochlear angle.

Measurement	Trochlear Angle ≤ 140° (*n* = 27)	Trochlear Angle > 140° (*n* = 23)	*p*-Value
TT-TG Distance (CT)	Mean: 2.04 cm (SD: 0.33)	Mean: 1.94 cm (SD: 0.23)	0.056
TT-IC Distance (CT)	Mean: 2.45 cm (SD: 0.32)	Mean: 2.38 cm (SD: 0.23)	0.184
TT-TG Distance (MRI)	Mean: 2.02 cm (SD: 0.36)	Mean: 1.94 cm (SD: 0.23)	0.149
TT-IC Distance (MRI)	Mean: 2.42 cm (SD: 0.35)	Mean: 2.38 cm (SD: 0.24)	0.425

**Table 3:** Association of TT-IC and TT-TG distances with trochlear facet asymmetry.

Measurement	With Asymmetry (*n* = 21)	Without Asymmetry (*n* = 29)	*p*-Value
TT-TG Distance (MRI)	Mean: 1.99 cm (SD: 0.20)	Mean: 1.99 cm (SD: 0.37)	0.51
TT-IC Distance (MRI)	Mean: 2.42 cm (SD: 0.21)	Mean: 2.40 cm (SD: 0.36)	0.984
TT-TG vs. TT-IC Difference (MRI)	Z = −4.018; *p* < 0.001	Z = −4.759; *p* < 0.001	

**Table 4:** Diagnostic performance of TT-TG and TT-IC distances using ROC analysis.

Parameter	AUC	Cut-Off Value	Sensitivity (%)	Specificity (%)	*p*-Value
TT-TG Distance	0.778	1.86 cm	78	62	0.001
TT-IC Distance	0.787	2.25 cm	85	62	<0.0001

## Data Availability

All the data processed in this article are part of the research for a doctoral thesis, which is archived in the pathology department at the University Hospital of Bucharest, where the interventions were performed. The original data are available upon reasonable request.
